# Neuropharmacology of the Neuropsychiatric Symptoms of Dementia and Role of Pain: Essential Oil of Bergamot as a Novel Therapeutic Approach

**DOI:** 10.3390/ijms20133327

**Published:** 2019-07-06

**Authors:** Damiana Scuteri, Laura Rombolà, Luigi Antonio Morrone, Giacinto Bagetta, Shinobu Sakurada, Tsukasa Sakurada, Paolo Tonin, Maria Tiziana Corasaniti

**Affiliations:** 1 Preclinical and Translational Pharmacology, Department of Pharmacy, Health Science and Nutrition, University of Calabria, 87036 Rende, Italy; 2 Department of Physiology and Anatomy, Faculty of Pharmaceutical Sciences, Tohoku Medical and Pharmaceutical University, 4-4-1 Komatsushima, Aoba-ku, Sendai 981-8558, Japan; 3 Daiichi College of Pharmaceutical Sciences—First Department of Pharmacology Fukuoka, Fukuoka 815-8511, Japan; 4 Regional Center for Serious Brain Injuries, S. Anna Institute, 88900 Crotone, Italy; 5 Department of Health Sciences, University “Magna Graecia” of Catanzaro, 88100 Catanzaro, Italy

**Keywords:** dementia, behavioral and psychological symptoms of dementia, neuropsychiatric symptoms, aromatherapy, bergamot essential oil

## Abstract

Aging of the population makes of dementia a challenge for health systems worldwide. The cognitive disturbance is a serious but not the only issue in dementia; behavioral and psychological syndromes known as neuropsychiatric symptoms of dementia remarkably reduce the quality of life. The cluster of symptoms includes anxiety, depression, wandering, delusions, hallucinations, misidentifications, agitation and aggression. The pathophysiology of these symptoms implicates all the neurotransmitter systems, with a pivotal role for the glutamatergic neurotransmission. Imbalanced glutamatergic and GABAergic neurotransmissions, over-activation of the extrasynaptic N-methyl-D-aspartate (NMDA) receptors and alterations of the latter have been linked to the development of neuropsychiatric symptoms experienced by almost the entire demented population. Drugs with efficacy and safety for prevention or long term treatment of these disorders are not available yet. Aromatherapy provides the best evidence for positive outcomes in the control of agitation, the most resistant symptom. Demented patients often cannot verbalize pain, resulting in unrelieved symptoms and contributing to agitation. Bergamot essential oil provides extensive preclinical evidence of analgesic properties. Incidentally, the essential oil of bergamot induces anxyolitic-like effects devoid of sedation, typical of benzodiazepines, with a noteworthy advantage for demented patients. These data, together with the reported safety profile, form the rational basis for bergamot as a neurotherapeutic to be trialed for the control of behavioral and psychological symptoms of dementia.

## 1. Introduction: Neuropsychiatric Symptoms of Dementia

The global impact of dementia is very serious, since 50 million people all over the world are affected and this figure is expected to triple within 2050 [1]. Among the different forms of dementia, Alzheimer’s disease (AD) is the most common and accounts for about two thirds of all cases [1]. The current AD pipeline includes 112 agents [2] and, while waiting for the discovery of disease-modifying drugs, the treatment of these patients is a very complex issue. Dementia is characterized by progressive deficits of memory, thinking, orientation, comprehension and communication, which are the main targets of the pharmacological action towards AD. Interestingly, quality of life (QoL) of patients is remarkably affected by disturbances of behavior, mood, thought content and perception [3,4,5,6,7]. This latter cluster of symptoms is known as Behavioral and Psychological Symptoms of Dementia (BPSDs) or Neuropsychiatric Symptoms (NPSs), early hallmarks of AD remarkably reducing the quality of life and affecting some 97% of demented patients, who experiences at least one of these fluctuating symptoms over the course of the disease, as assessed in the Cache County Study [8]. There are several syndromes according to the experienced NPSs that can include irritability, anxiety, depression, apathy, agitation, aggression, psychotic symptoms as hallucination, aberrant motor behavior, disinhibition, elation etc. The occurrence of NPSs can be prodromal to the onset of dementia and often induces institutionalization. The time course of these syndromes was studied in volunteers at National Institute on Aging–funded Alzheimer’s Disease Centers [9]. It has been shown that NPSs occur earlier than dementia in most cases for all types of dementia and mild cognitive impairment (MCI). The 5-year longitudinal cohort study “Dementia Study of Western Norway (Demvest)” reported a mean decline of the Mini-Mental State Examination (MMSE) by 2.1 points/year and, in contrast, a median slight increase of the Neuropsychiatric Inventory (NPI) score from 15 at baseline to 17 at year 5 [10]. The symptoms most likely present in cognitive decline were delusions, hallucinations, agitation, apathy and aberrant motor behavior in NPI [10]. Interestingly, the 97% (i.e., almost the whole sample) displayed an NPI total score ≥16 ever and the 49% had ≥36 ever, which stands for need of pharmacological antipsychotic treatment in trials [10]. Agitation is one of the most challenging symptoms of which the principal features are excessive motor activity or verbal or physical aggression [11]. It is predictive of worse prognosis and increased risk of injury. The prevalence of agitation/aggression in AD was observed to be 47.38% [12]. Growing evidence suggests that these symptoms do not depend on cognitive impairment exclusively, since they could be due to peculiar neurotransmitter dysfunctions [13,14].

## 2. Neuropharmacology of NPSs

The diverse NPSs are characterized by macroscopic and microscopic neuropathological lesions typical of dementia in different anatomic areas, thus a comprehension of this pathogenesis is needed [15]. Neurofibrillary tangles were extensively found in amygdala [16], basal nucleus of Meynert [17], dorsal raphe nucleus [18] and locus coeruleus with following neurodegeneration of the originating noradrenergic projections [19]. Imaging studies revealed that BPSDs are associated with lower metabolism and perfusion in the frontal and temporal lobes. Moreover, a large amount of neurofibrillary tangles has been found concurrently with agitation and psychosis (see reference [20]). Neuropsychiatric symptoms were related to hypometabolism of different regions: e.g., psychosis to frontal cortical, agitation/disinhibition to temporal cortical and anxiety/depression to parietal cortical, respectively [21,22]. Moreover, the pathophysiological features differ based on the type of NPS: for instance, disinhibition, apathy, and frontal dysfunction in AD are linked to excess of neurofibrillary tangles in the frontal lobes, while visual hallucinations and delusions often occur in dementia with Lewy bodies [23]. Despite these associations, not all the patients affected by a specific form of dementia develop the same NPSs [23]. According to analysis of the dorso-lateral prefrontal cortex (Brodmann area 9—BA9), anterior cingulate gyrus (BA24) and parietal cortex (BA40) delusions and agitation results are significantly linked to Tau tangle pathology and negatively correlated with the levels of the synaptic vesicle zinc transporter ZnT3 [24]. 

Several neurotransmitters have been implicated in the development of these behavioral neuropsychiatric syndromes. A variable lack of balance among the several neurotransmissions may be involved in NPSs development and could explain the fluctuant nature of these syndromes [25].

Cholinergic deficits mainly in the fronto-temporal lobes are involved in diverse neuropsychiatric manifestations of dementia, like delusions of burglary and infidelity or misidentification, as well as of several psychotic conditions (see reference [26]). A role of the cholinergic system in agitation has been hypothesized since anticholinergic drugs increase agitation, while cholinergic agents reduce this symptom (see reference [26]). An α2-adrenergic receptor binding study reported a 70% increase of these receptors in AD patients suffering from agitation and aggression in comparison with the not aggressive patients [27]. Aggression and the treatment with antipsychotics in AD were found to be linked to increased α1-adrenergic receptors in the dorsolateral prefrontal cortex [28,29], where there is an enhanced binding to α2-adrenergic receptors, as well as in middle temporal gyrus [29,30]. 

Dopamine has been hypothesized to take part in the integration of some behavioral aspects via the meso-limbic system; aggression likely seems to be linked to impairment of dopaminergic pathways [31]. Decreased levels of dopamine were detected in the cingulate gyrus, amygdala, striatum, raphe nuclei and cerebrospinal fluid in AD [29,32,33]. Furthermore, a reduction of striatal D2 receptors in patients suffering from AD was associated with more severe BPSDs [22,34]. 

Also, serotonin (5-HT) reduction in AD has been implicated in the development of BPSDs. In particular, the different behavioral syndromes characteristic of each patient may be due to an imbalance of more neurotransmitter systems [31]. Cellular alterations of neurons in the raphe nuclei of AD brains were reported: frequent features shown were globose neurofibrillary tangles in the perikaryon and a significant decrease of nucleolar volume and cytoplasmic RNA in medial and lateral dorsal tegmental nucleus [35]. Reduced levels of 5-HT and of 5-hydroxyindoleacetic acid in temporal cortex mainly were highlighted (see reference [36]). 

By contrast, the role of γ-aminobutyric acid (GABA) in the presentation of NPSs is not fully understood yet (see reference [36]). An imbalance between glutamatergic and GABAergic transmissions, in circuits already more susceptible because of acetylcholine deficiency, was tested as possibly being involved both in cognitive decline and in neuropsychiatric manifestations [14]. No significant differences in glutamate content in BA10 and BA20 of AD brains were demonstrated, while GABA concentrations were significantly reduced by 21%; although there was no correlation between glutamate content and BPSDs, the ratio glutamate/GABA resulted in the best predictor for the depression factor score in BA10 for AD patients [14]. Also, an imbalance between cholinergic and serotonergic systems is involved in NPSs: the best predictor of lowered ChAT and AChE levels both in BA10 and BA20 was the aggression score and the ratio AChE/5-HT was the best predictor for the psychotic factor, as demonstrated for women [37]. 

Another interesting finding is that SLC6A4, the gene encoding 5-HT transporter, is subjected to several polymorphisms affecting its expression. In particular, 5-HTTVNTR allele 10 was associated with BPSDs and aggression [38,39]. The 5-HT2A T102C polymorphism has been proposed as a predisposing factor to BPSDs in AD patients at the transcriptional or posttranslational level [40]. Furthermore, it seems to be correlated to a decrease of 5-HT2A receptors in the temporal cortex [41], thus impairing serotonergic modulation of the dopaminergic pathways and, likely, inducing the psychosis spectrum [42,43].

### Glutamatergic Transmission and NPSs

The NPSs typical of dementia are characterized by neuropharmacological alterations of the main neurotransmissions, variously investigated but not completely unraveled. The neurochemical correlates of these behavioral syndromes in the cerebrospinal fluid were investigated through lumbar puncture: the sample was searched for the amino acids aspartate, glutamate, glutamine, glycine, taurine, and proline and for norepinephrine, dopamine, 3,4-dihydroxyphenylacetic acid and 5-hydroxyindoleacetic acid at ultraperformance liquid chromatography, whereas for homovanillic acid at high-performance liquid chromatography [44]. According to the results:

- patients affected by AD showed a positive correlation of the ratio homovanillic acid/5-hydroxyindoleacetic acid with the cluster anxieties/phobias as assessed through the BEHAVE-AD;
- patients with dementia with Lewy bodies were found to show a negative correlation between homovanillic acid and the cluster hallucinations at BEHAVE-AD;
- taurine was inversely correlated with the Cornell Scale for Depression and BEHAVE-AD;
- patients suffering from frontotemporal dementia presented an inverse correlation of glutamate with the cluster verbally agitated behavior at the Cohen–Mansfield Agitation Inventory [44].

Glutamate may play a fundamental role in dementia-related agitation and anxiety because it is the major excitatory neurotransmitter. A study conducted at the University of California, Los Angeles Alzheimer Disease Research Center (UCLA-ADRC) highlighted:

- an increase of the binding affinity to glycine recognition site;
- a reduction of NR2A subunits compared to NR2B of N-methyl-D-aspartate (NMDA) receptors in the postmortem orbitofrontal cortex of AD patient subgroups with higher anxiety [45].

An altered balance between the activity of synaptic and extrasynaptic NMDA receptors with over-activation of the extrasynaptic component in subgenual cingulate region BA25 area has been proposed as the mechanism at the root of glutamate-based depression [46]. The involvement of the extrasynaptic NMDA receptors, responsible for the activation of pathways prompting synaptic damage, is a key feature of AD. This is demonstrated by the use of memantine, which exerts neuroprotection via an uncompetitive/fast-off rate acting mainly on these extrasynaptic receptors [47]. 

Social isolation housing is a model of BPSD-like behavioral disturbances in rodents. The intracerebroventricular injection of β amyloid 1-42 in isolated 11 week-old mice induced aggressive behavior in the resident-intruder test and anxiety behavior in the plus-maze test, according to reduction of the time spent in open arms 3 weeks after injection [48]. Moreover, an increase of the serum levels of corticosterone and an enhancement of presynaptic activity in the Schaffer collateral-CA1 pyramidal cell and in the mossy fiber-CA3 pyramidal cell synapses exist [48]. The latter findings may stand for a corticosterone-induced increase of hippocampal glutamatergic activity, likely implicated in these NPS-like syndromes [48]. 

## 3. Novel Pharmacological Mechanisms for NPSs of Dementia Clinical Management: The Essential Oil of Bergamot

A treatment for dementia NPSs endowed with efficacy and safety still remains a serious challenge. An extrapolation of the effects of antipsychotics in the treatment of primary neuropsychiatric disturbances led to their off label use also in BPSDs, regardless of the differences likely occurring between primary and secondary disorders in terms of neuropsychopathology and, consequently, of effectiveness and safety of the drug [49]. Based on the most expressed NPS in the individual syndrome, other pharmacological agents include antidepressants, methylphenidate, benzodiazepines, zolpidem, Z-agents etc [49]. The atypical antipsychotics, such as risperidone, olanzapine, aripiprazole and quetiapine, are more used than the typical agents because of the improved pharmacodynamic profile useful for the treatment of schizophrenia, but the increased risk of death for cerebrocardiovascular side effects is a serious issue in demented patients. Risperidone is considered the safest in the management of NPSs for short-term with accurate review of the treatment [50]. 

Because of the involvement of multiple neurotransmissions in NPSs, a novel investigational drug, lumateperone tosylate, is a first-in-class agent under study along with several mechanisms for the treatment of agitation in dementia [51]:

- antagonist at 5-HT2A receptors;
- partial agonist at presynaptic D2 receptors, while antagonist at postsynaptic receptors;
- enhancer of NMDA and α-amino-3-hydroxy-5-methyl-4-isoxazolepropionic acid (AMPA) receptor activity through the pathway of the mammalian target of rapamycin (mTOR).

Actually, pimavanserin, a selective 5-HT2A receptor inverse agonist and antagonist, is endowed with efficacy in psychosis associated to AD, though only up to the 6th week of treatment [52]. 

**Figure 1:**
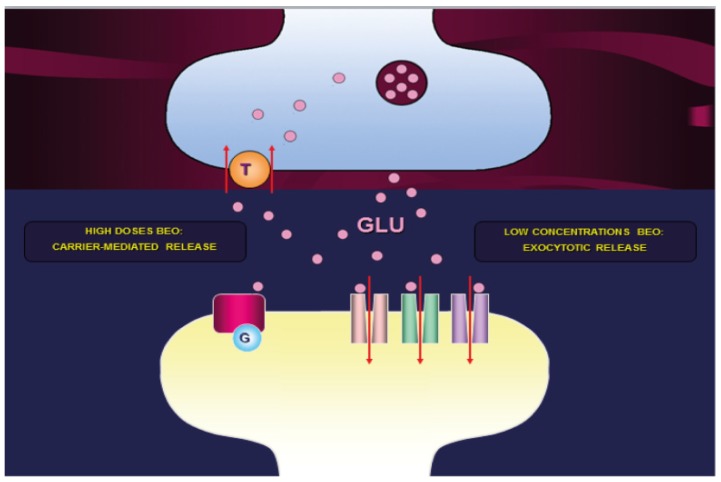
Mechanism of BEO-induced increase of synaptic glutamate. BEO in low concentrations causes glutamate exocytosis, while in high concentrations it induces the release of glutamate through a carrier-mediated Ca^2+^-independent process [58].

Aromatherapy, a specialized form of phytotherapy that uses essential oils, is a complementary treatment that has provided preliminary though promising evidence for the management of agitation in dementia [53]. A placebo-controlled trial on seventy-two care facilities residents suffering from dementia showed that massage applying Melissa officinalis essential oil reduces the score of agitation at the Cohen-Mansfield agitation inventory, without the occurrence of significant side effects [54]. Bergamot essential oil (BEO) was demonstrated to exert anxiolytic-like activity in animal behavioral tests [55]. In particular, BEO decreased grooming behavior in the open field test as diazepam and most of the anxiolyitic drugs, but without the loss of vigilance induced by diazepam [55]. This effect of BEO in the open field test was not significantly counteracted by flumazenil, hence it is not superimposable to the activity of benzodiazepines [56]. Indeed, BEO was observed to increase alpha electroencephalographic frequency of relaxation and beta brainwave activity of alert [57]. In support of the latter anxyolitic-like relaxant effect, BEO also increased the time spent in open arms in the elevated plus maze and immobility in the forced swimming test, a parameter that can suggest successful coping to stress in this task of evaluating antidepressant effects [55]. Therefore, aromatherapy using BEO can improve BPSDs and these effects could be due to the capability of this essential oil of increasing the levels of aspartate, glycine and taurine in a Ca^2+^-dependent manner after systemic administration and of synaptic glutamate and GABA in a Ca^2+^-independent manner through microdialysis in the hippocampus [58]. Moreover, BEO was demonstrated to foster the release of endogenous glutamate and pre-loaded [^3^H]D-aspartate concentration-dependently in hippocampal synaptosomes of rat, which was inhibited by the selective non-transportable inhibitor of excitatory amino acid transporters DL-threo-β-benzyloxyaspartic acid [58]. Hence, low concentrations of BEO can cause glutamate exocytosis, whereas high concentrations glutamate release via a carrier-mediated Ca^2+^-independent mechanism (Figure 1) [58]. 

Therefore, low concentrations of BEO may prompt the presynaptic exocytosis of glutamate, which can act on Gq-coupled group I metabotropic glutamate autoreceptors or heteroreceptors [59]. This mechanism can induce modulation of multiple neurotransmissions dysregulated in the neuropsychiatric symptoms of dementia and initiate retrograde endocannabinoid signaling responsible for disinhibition of the descending analgesic pathway acting on GABAergic interneurons [60]. 

Patients affected by dementia often present alteration of the nociceptive transduction and modulation pathways, as well as age-related comorbidities responsible for chronic pain often underdetected and mistreated because of their impaired communication skills [60]. Chronic non-cancer pain (i.e., low back pain, diabetic neuropathy, osteoarthritis and migraine), common in neurodegenerative disorders, represents a remarkable social burden [61]. For instance, migraine is often disabling because several following or concomitant stages of the disease can undergo chronification [62] and patients over 65 are not recommended for treatment with triptans [63]. The development of NPSs, and of agitation mainly, is linked to misdiagnosed [64,65] and unrelieved pain [66,67]. In fact, the demented patients who are provided with analgesic therapy are less common than the general population [68], thus supporting pain undertreatment that has been also highlighted in the local context [69,70]. It was demonstrated that a stepwise protocol for pain treatment significantly reduced agitation of the 17%, with an increase after withdrawal [71]. Therefore, aromatherapy using an essential oil endowed with strong analgesic properties could be even more useful in the management of BPSDs as agitation [72]. BEO has been proven to exert analgesic activity both in inflammatory [73,74,75] and in neuropathic [76,77] pain models, as well as via an inhalatory route of administration [78]. The analgesic properties of BEO are reported in Table 1.

**Figure 2:**
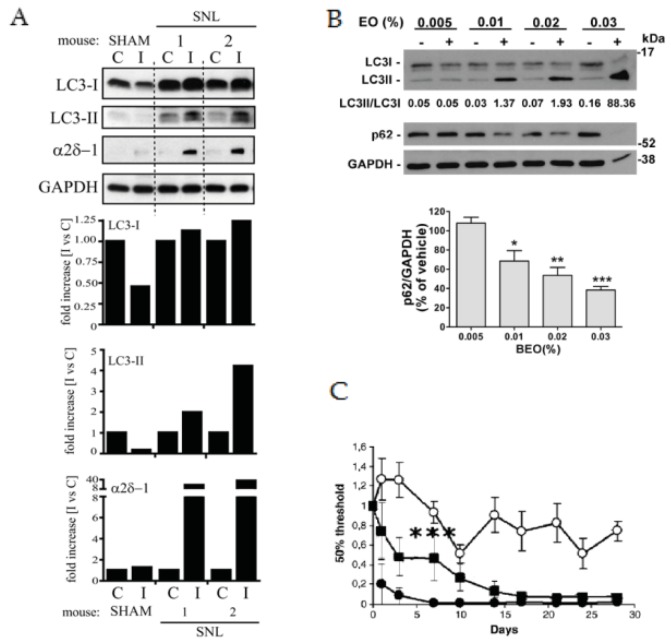
Autophagy and analgesic activity of BEO. (**A**) LC3 expression in the hemi-cord contralateral (C) and ipsilateral (I) to the side of ligation, 7 days after Spinal Nerve Ligation (SNL), showing higher LC3-I expression in ipsilateral side of SNL mice and appearance of LC3-II, thus demonstrating a derangement of autophagy in this neuropathic pain model. The slight increase in LC3-I levels and the apparent formation of LC3-II well correlated with α2δ-1 upregulation (Sham: *n* = 5, SNL: *n* = 6; adapted with permission from reference [81]). (**B**) BEO-mediated concentration-dependent induction of autophagy in SH-SY5Y cells, demonstrated by immunoblot showing the conversion of LC3I to LC3II and reduced p62 levels. Histogram shows the densitometric analysis of p62 levels normalized on the values of GAPDH (used as loading control) expressed as percentage of vehicle from three independent experiments (mean ± SEM). * *p* < 0.05, ** *p* <0.01, *** *p* < 0.001 vs. 0.005% BEO (ANOVA followed by Tukey-Kramer multiple comparisons test; adapted with permission from reference [84]). (**C**) A daily dose of BEO (square; 1 mL/kg) subcutaneously administered for 7 days attenuated SNL-induced mechanical allodynia compared to vehicle (filled circles; *** *p* < 0.001). Open circles indicate mechanical sensitivity of sham operated mice. Data are expressed as mean ± SEM of 50% of pain threshold and normalized to the basal value of each animal (*n* = 5–10 per group). Differences are evaluated using one way analysis of variance (ANOVA), followed by Tukey multiple comparisons test. Adapted with permission from reference [76].

It is important to underline that aromatherapy for inhalation would be effective by virtue of the systemic absorption of BEO eliciting its pharmacological action but not because of a psychological perception of the fragrance [78], since patients suffering from dementia may be anosmic [79]. Glutamatergic modulation can explain also the analgesic properties of BEO [60]. Glutamate at the first synapse is implicated in central sensitization, and in modulation of painful stimuli through the metabotropic receptors involved in the release of endogenous opioid peptides and endocannabinoids [60], able to activate type 1 vanilloid receptor TRPV1 and to modulate the release of different neurotransmitters [80]. Glutamate induced pain sensitization can implicate derangement of autophagy, a process subjected to derangement in neuropathic pain [81]. Indeed, modulation of autophagy induced by glutamate has been investigated in vitro [82]. Glutamate induced autophagy, was accompanied by an increase of signals of LC3-II, as NAADP (nicotinic acid adenine dinucleotide phosphate) [82], which has been hypothesized to be a second messenger of glutamate [82,83]. In particular, glutamate fostered the mobilization of Ca^2+^ in a manner that depends on NAADP-regulated channels [82]. Moreover, pre-treatment of cells with the antagonist of the lysosomal Ca^2+^-permeable two-pore channels, i.e., NED-19, failed to increase basal levels of LC3-II, while no further increase occurred with glutamate [82]. This suggests that induction of autophagy by glutamate is inhibited by NED-19 and induced through NAADP and the NAADP-sensitive Ca^2+^-permeable two-pore channels; accordingly, silencing of the latter channels prevented the glutamate-induced increase of autophagy in astrocytes and SH-SY5Y cells [82]. This glutamate-induced autophagic flux via NAADP has been suggested to be linked to AMP-activated protein kinase pathway, a Ca^2+^ and energy deprivation responsive upstream modulator of autophagy [82]. Incidentally, BEO-induced analgesia implicates the induction of basal and induced autophagy [84], as illustrated in Figure 2. 

Clinical trials that are able to clear any doubt and to provide sound basis for the use of phytotherapeutic interventions with essential oils (i.e., aromatherapy) in the management of dementia are needed [85]. The evidence accumulated so far supports the need for a rigorous clinical trial in patients affected by dementia, in order to assess efficacy and safety of aromatherapy with BEO in the management of the several neuropsychiatric behavioral syndromes related to this neurodegenerative disorder [86].

## Figures and Tables

**Table 1:** Effects of BEO in experimental models of pain.

Analgesic Effect	Pain Model	Route of Administration	Main Results of the Research	Study
Antinociceptive effect on licking/biting response	Capsaicin test [73,74]	Intraplantar [73]	BEO (5, 10 and 20 mg) exerted antinociceptive effect in the capsaicin test (50 µg) [73].	Sakurada et al., 2009 [73]
		Subcutaneous into the plantar surface [74]	BEO (20 μg) produced significant antinociception in capsaicin test (1.6 μg), only in the ipsilateral side, reverted by naloxone hydrochloride and methiodide, suggesting a role of peripheral opioid system [74]	Sakurada et al., 2011 [74]
	Formalin test [75,78].	Plantar subcutaneous [75]	BEO (10 μg) significantly inhibited the nociceptive response to 2% formalin, only in the ipsilateral side, and this effect was antagonized by naloxone hydrochloride and methiodide [75]	Katsuyama et al., 2015 [75]
		Inhalatory [78]	A filter paper disc soaked with different volumes of BEO (100, 200, 400, 800 μL) to the edge of the cage allowed inhalation of BEO in different experimental settings, showing its antinociceptive activity in formalin test (2%) in a volume and time of exposure dependent manner [78]	Scuteri et al., 2018 [78]
Antiallodynic effect	Spinal nerve ligation [76]	Subcutaneous into the plantar surface [76]	BEO (1 mL/kg) subcutaneously administered daily for 7 days attenuated mechanical allodynia [76]	Bagetta et al., 2010 [76]
	Partial sciatic nerve ligation [77]	Subcutaneous into the plantar surface [77]	On post-operative day 7, BEO (5.0, 10.0 and 20.0 μg) dose-dependently increased ipsilateral hindpaw withdrawal thresholds and blocked spinal ERK activation [77].	Kuwahata et al., 2013 [77]

## Abbreviations

AD	Alzheimer’s disease
AMPA	α-amino-3-hydroxy-5-methyl-4-isoxazolepropionic acid
BPSDs	Behavioral and Psychological Symptoms of Dementia
BA	Brodmann area
BEO	Bergamot essential oil
CNS	Central Nervous System
GABA	γ-aminobutyric acid
mTOR	mammalian target of rapamycin
MCI	Mild cognitive impairment
MMSE	Mini-Mental State Examination
NAADP	Nicotinic acid adenine dinucleotide phosphate
NMDA	N-methyl-D-aspartate
NPI	Neuropsychiatric Inventory
NPSs	Neuropsychiatric Symptoms
QoL	Quality of life
5-HT	Serotonin
ZnT3	Synaptic vesicle zinc transporter
TRPV1	Type 1 vanilloid receptor
UCLA-ADRC	University of California, Los Angeles Alzheimer Disease Research Center
